# Protection against collagen-induced arthritis in mice afforded by the parasitic worm product, ES-62, is associated with restoration of the levels of interleukin-10-producing B cells and reduced plasma cell infiltration of the joints

**DOI:** 10.1111/imm.12208

**Published:** 2014-02-10

**Authors:** David T Rodgers, Miguel A Pineda, Mairi A McGrath, Lamyaa Al-Riyami, William Harnett, Margaret M Harnett

**Affiliations:** 1 Institute of Infection, Immunity and Inflammation, University of GlasgowGlasgow, UK; 2 Strathclyde Institute of Pharmacy and Biomedical Sciences, University of StrathclydeGlasgow, UK

**Keywords:** ES-62, interleukin-10-producing B cells, parasitic helminths, rheumatoid arthritis

## Abstract

We have previously reported that ES-62, a molecule secreted by the parasitic filarial nematode *Acanthocheilonema viteae*, protects mice from developing collagen-induced arthritis (CIA). Together with increasing evidence that worm infection may protect against autoimmune conditions, this raises the possibility that ES-62 may have therapeutic potential in rheumatoid arthritis and hence, it is important to fully understand its mechanism of action. To this end, we have established to date that ES-62 protection in CIA is associated with suppressed T helper type 1 (Th1)/Th17 responses, reduced collagen-specific IgG2a antibodies and increased interleukin-10 (IL-10) production by splenocytes. IL-10-producing regulatory B cells have been proposed to suppress pathogenic Th1/Th17 responses in CIA: interestingly therefore, although the levels of IL-10-producing B cells were decreased in the spleens of mice with CIA, ES-62 was found to restore these to the levels found in naive mice. In addition, exposure to ES-62 decreased effector B-cell, particularly plasma cell, infiltration of the joints, and such infiltrating B cells showed dramatically reduced levels of Toll-like receptor 4 and the activation markers, CD80 and CD86. Collectively, this induction of hyporesponsiveness of effector B-cell responses, in the context of the resetting of the levels of IL-10-producing B cells, is suggestive of a modulation of the balance between effector and regulatory B-cell responses that may contribute to ES-62-mediated suppression of CIA-associated inflammation and inhibition of production of pathogenic collagen-specific IgG2a antibodies.

## Introduction

The prevalence of autoimmune disease in the developing world inversely correlates with parasitic helminth infection,^1^,^2^ an apparent serendipitous side-effect of the ability of worms to secrete anti-inflammatory molecules that modulate the host immune system and promote parasite survival. Such epidemiological evidence has understandably generated interest in the therapeutic potential of such immunomodulators and also in their use as tools to dissect the pathogenic mechanisms underlying inflammatory disorders.^3^ We have previously shown that one such immunomodulator, ES-62, a phosphorylcholine-containing glycoprotein, secreted by the filarial nematode *Acanthocheilonema viteae*, is protective in the mouse collagen-induced arthritis (CIA) model of rheumatoid arthritis (RA) by acting to suppress pathogenic T helper type 1 (Th1)/Th17 responses.^4^ The success of rituximab as a therapy for autoimmune disease has refocused interest on the pathogenic and protective roles of B cells in RA^5^ with recent studies highlighting the importance of interleukin-10 (IL-10)-producing regulatory B (Breg) cells in the prevention and amelioration of CIA and also antigen-induced arthritis (AIA), via the suppression of Th1/Th17 responses and the promotion of type 1 regulatory T (Tr1) and regulatory T (Treg) cell differentiation.^5^–^8^ Interestingly, therefore, we have found that while exposure to ES-62 results in enhanced spontaneous *ex vivo* production of IL-10 by splenocytes from mice with CIA, it induces hyporesponsiveness of normal and CIA-derived splenic B cells and reduces the levels of pathogenic collagen-specific IgG2a antibodies.^9^ Relating to this, we now show that the protective effects of ES-62 in CIA are associated with restoration of the levels of IL-10-producing B cells and suppression of the infiltration of activated plasma cells into the joints, perhaps suggesting that ES-62 may act, at least in part, to modulate the balance between effector and regulatory B-cell responses in this mouse model of RA.

## Materials and methods

### Collagen-induced arthritis

Animals were maintained in the Biological Services Units in accordance with the Home Office UK Licences PPL60/3580, PPL60/3791, PPL60/4300 and PIL60/9576, PIL60/12183 and PIL60/12950 and the Ethics Review Boards of the Universities of Glasgow and Strathclyde. Arthritis was induced in male DBA/1 mice (8–10 weeks old; Harlan Olac, Bicester, UK) by intradermal immunization with bovine type II collagen (MD Biosciences, Zurich, Switzerland) in complete Freund's adjuvant on day 0 and in PBS on day 21. Mice with CIA were treated with purified endotoxin-free ES-62 (2 μg/dose) or PBS subcutaneously on days −2, 0 and 21 and cells were recovered from joints^10^ as previously described.^4^,^9^,^11^ All analysis was performed at cull (day 28) and represents data from at least two independent experiments.

### *Ex vivo* analysis

**Figure 1:**
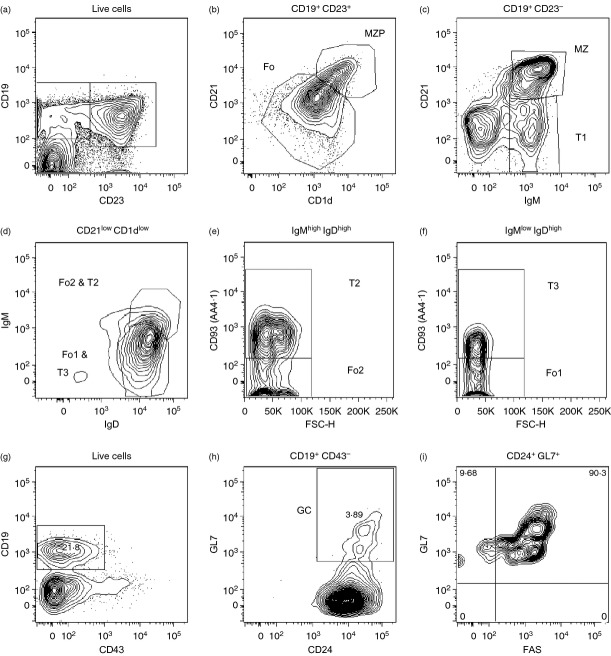
Gating strategy for analysis of B-cell subsets and phenotyping of populations. This is a modification of that based on the peripheral B cell phenotypic markers defined by Allman and Pillai.^14^ T1: CD19^+^ CD93^+^ CD21^int^ CD23^−^ IgD^low/−^ IgM^high^; T2: CD19^+^ CD93^+^ CD21^int^ CD23^+^ IgD^high^ IgM^high^; T3: CD19^+^ CD93^+^ CD21^int^ CD23^+^ IgD^high^ IgM^low^; marginal zone precursor (MZP): CD19^+^ CD93^−^ CD21^high^ CD23^+^ CD1d^high^ IgD^high^ IgM^high^; marginal zone (MZ): CD19^+^ CD93^−^ CD21^high^ CD23^*−*^ CD1d^high^ IgD^low/*−*^ IgM^high^; Fo1: CD19^+^ CD93^*−*^ CD21^low^ CD23^+^ IgD^high^ IgM^low^; Fo2: CD19^+^ CD93^*−*^ CD21^low^ CD23^+^ IgD^high^ IgM^high^; GC: CD19^+^ CD43^*−*^ CD24^+^ GL7^+^. Cell populations were initially selected on the basis of ‘Lymphocyte’ size (forward scatter; FSC) and granularity (side scatter; SSC) parameters and excluding ‘doublets’ (by comparing FSC-Height and FSC-Area) and dead cells (by the Live/Dead® fixable aqua dead cell dye; Invitrogen) (data not shown). We initially gated on CD19^+^ CD23^*−*^ and CD19^+^ CD23^+^ cells (a) to resolve MZP (CD21^high^ CD1d^high^) from follicular (Fo) (CD21^low^ CD1d^low^) B cells (b) and MZ (CD21^+^ IgM^+^) and T1 (CD21^*−*^ IgM^+^) cells (c), respectively. The Fo population identified (b; CD21^low^ CD1d^low^) is a heterogeneous population that contains the functionally distinct follicular type 1 (Fo1: IgD^high^ IgM^low^ AA4·1^−^) and follicular type 2 (Fo2: IgD^high^ IgM^high^ AA4·1^−^) as well as the transitional 2 (T2: IgD^high^ IgM^high^ AA4·1^+^) and transitional 3 (T3: IgD^high^ IgM^low^ AA4·1^+^) populations. These populations are first separated on the basis of their expression of IgM and IgD (d) and then, AA4·1 (e,f). For the identification of germinal centre (GC) B cells we first identify CD19^+^ CD43^*−*^ cells (g) and then exclude contaminating non-B cells by gating on the GC cell-specific marker GL7 along with the pan-B-cell marker CD24 (h) before confirming expression of FAS (i) by essentially all (> 90%) CD19^+^ CD43^*−*^ CD24^+^ GL7^*+*^ GC B cells; we have therefore not included this redundant marker in our analysis.

Splenocytes and draining lymph node (DLN) cells (10^6^/ml) were analysed for B-cell IL-10 responses by stimulating with or without 50 ng/ml PMA (Sigma-Aldrich, Poole, UK) plus 500 ng/ml ionomycin (Sigma-Aldrich) and 10 μg/ml lipopolysaccharide (*Escherichia coli* O111:B4; Sigma-Aldrich) for 1 hr before addition of 10 μg/ml brefeldin A (Sigma-Aldrich) for 5 hr at 37° with 5% CO_2_.^12^,^13^ Lymphocyte subsets were analysed by flow cytometry of unstimulated cells adapting the gating strategy (Fig. 1) of Allman and Pillai^14^ using antibodies specific for the following markers (with relevant fluorochrome): CD5/Biotin-svE450; CD8/Biotin-sv peridinin chlorophyll protein streptavidin (svPerCP) (both BD Pharmingen, Franklin Lakes, NJ); AA4.1/allophycocyanin (APC); B220/BV421; CD11c/Biotin-svPerCP; CD138/phycoerythrin (PE); CD19/AF700; CD1d/PE; CD23/PE-Cy7; CD24/PerCP-Cy5.5; CD4/Biotin-svPerCP; CD43/PE-Cy7; IgD/PerCP-Cy5.5; IgM/APC-Cy7; F4/80/Biotin-svPerCP (all BioLegend, San Diego, CA), and CD21/E450 and GL7/E450 (both eBioscience, San Diego, CA). Additional phenotypic markers were labelled using anti-Toll-like receptor 4 (TLR4)-APC (R&D Systems, Abingdon, UK), anti-BAFF-R-FITC (eBioscience), anti-CD4-PE, anti-CD80-PerCP/Cy5.5 or anti-CD86-AF488 (BioLegend) antibodies before the cells were fixed and permeabilized using BioLegend products and protocols. Stimulated cells were then labelled using anti-IL-10-APC (BioLegend) antibodies for 30 min before flow cytometry to detect IL-10-producing B cells. Data analysis gates were set according to appropriate isotype controls. Dead cells were identified and excluded from analysis using the Live/Dead® Fixable Dead Cell Stain (Aqua) using the manufacturer's suggested protocol (Invitrogen, Paisley, UK).

### Statistics

Parametric data were analysed by the Student's *t*-test or by one-way analysis of variance (anova) while non-parametric data were analysed by Mann–Whitney and Kruskal–Wallis tests where *P < 0·05, **P < 0·01 and ***P < 0·001.

## Results

### ES-62 reduces the levels of germinal centre (GC) B cells in the spleens of mice with CIA

**Figure 2:**
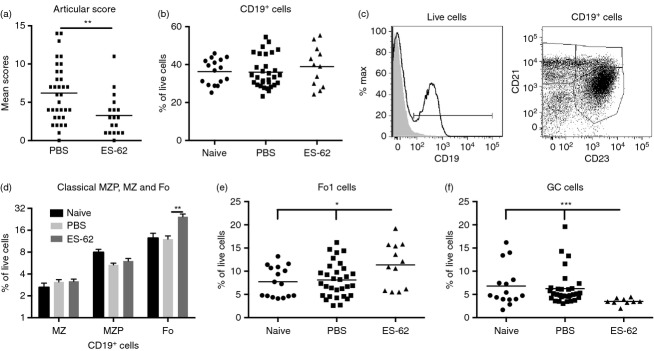
ES-62 reduces the levels of germinal centre B cells in the spleens of mice with collagen-induced arthritis (CIA). Mean articular scores (±SEM) of CIA mice treated with PBS (*n *= 34) or ES-62 (*n *= 18) at time of cull at day 28 (a). The percentage of CD19^*+*^ B cells (b); representative plots (c) and proportions (d; mean values ± SEM of individual mice where naive, *n *= 16; PBS, *n *= 31; ES-62, *n *= 12) of marginal zone precursor (MZP), marginal zone (MZ) and follicular (Fo) B cells as defined by their expression of CD21 and CD23; Follicular type 1 B cells (Fo1, e: CD19^*+*^ CD23^*+*^ CD21^low^ CD1d^low^ IgM^low^ IgD^high^ AA4·1^*−*^) and germinal centre cells (GC; f: CD19^*+*^ CD43^*−*^ CD24^*+*^ GL7^*+*^), as derived by the gating strategy presented in Fig. 1, in spleens from mice undergoing CIA are shown.

We investigated whether ES-62-mediated protection against CIA (Fig. 2a) correlated with modulation of B-cell populations (Fig. 1). There was no significant modulation by the helminth product of either the proportion or number of CD19^+^ B cells in the spleen [Fig. 2b; numbers (× 10^6^) ± SEM: Naive, 25·13 ± 2·26; PBS, 26·86 ± 1·76; ES-62, 28·27 ± 2·68] or LN (data not shown) and consistent with this, no significant changes were observed in the transitional (T1–T3), marginal zone precursor or marginal zone populations in the spleen (Fig. 2c,d and results not shown). However, ES-62 significantly increased the levels of CD19^+^ CD21^low^ CD23^high^ follicular B cells (Fo; Fig. 2c,d) and further analysis showed that although exposure to ES-62 had no effect on follicular type-2 (Fo2) B cells (results not shown), it significantly increased the levels of follicular type-1 B2 cells (Fo1; Fig. 2e; numbers (× 10^6^) ± SEM: Naive, 5·58 ± 0·82; PBS, 6·08 ± 0·64; ES-62, 7·97 ± 0·91) in the spleen. The increase in Fo1 B cells was associated with corresponding reductions in GC B cells (Fig. 2f; numbers (× 10^6^) ± SEM are: PBS, 6·28 ± 1·36; ES-62, 2·95 ± 0·39) and CD3^+^ CD4^+^ ICOS^+^ CXCR5^+^ follicular helper T cells [from 2·42 (PBS) to 1·49% of live splenocytes exposed to ES-62] in the spleen. By contrast, ES-62 had no effect on the levels of CD19^+^ B220^+^ IgM^−^ IgD^−^ IgG^+^ cells, which may represent a subset of memory B cells (results not shown). Collectively, these data suggest that ES-62 may act to reduce the generation of pathogenic antibodies by blocking the activation of follicular B cells and their consequent differentiation into GC B cells.^15^

### ES-62 modulates the recruitment of B cells to the joints of mice with CIA

**Figure 3:**
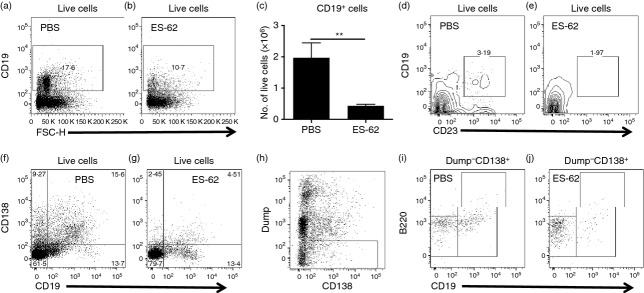
ES-62 modulates the recruitment of B cells to the joints of mice with collagen-induced arthritis (CIA). Cells extracted from the joints of mice with CIA were analysed for the proportion (a,b) and number (c) of infiltrating CD19^+^ B cells (data in c are presented as the means ± SEM of four biological replicates pooled from two independent experiments) and consequently for the relative proportions of CD19^+^ CD23^+^ (d,e) and plasma cells on the basis of CD19 CD138 expression (f,g). Exclusion of myeloid and T-cell-expressing CD138 cells by use of Dump channel (CD4^+^ CD8^+^ GR1^+^ F4/80^+^ CD11b^+^ CD11c^+^; h) allowed analysis of Dump^−^ CD19^−^ B220^−^ CD138^+^ and Dump^−^ CD19^+^ B220^low/−^ CD138^+^ plasma cells (i,j).

As pathogenic B cells migrate to the joints and even form ectopic GCs in response to B-cell recruitment and survival factors such as BAFF, CXCL12 and CXCL13 secreted by synovial fibroblasts,^5^ we next analysed whether the above effects of *in vivo* exposure to ES-62 on the profile of B cells were reflected in the arthritic joint. This revealed that both the proportion (Fig. 3a,b) and absolute numbers (Fig. 3c) of CD19^+^ B cells found in the joints were significantly reduced by ES-62 treatment. This reduction was reflected in a CD19^+^ CD23^+^ B-cell population (Fig. 3d,e), which further analysis revealed to be Fo1 B cells (Table 1). There was also a clear decrease in CD19^−^ CD138^+^ (from 9·27 to 2·45% live cells) and CD19^+^ CD138^+^ (from 15·6 to 4·51% live cells) cells infiltrating the joints of mice treated with ES-62 (Fig. 3f,g), which suggested a reduction in plasma cells. Consistent with this, further analysis, excluding the myeloid and T-cell lineages expressing CD138 (Fig. 3h), revealed that exposure to ES-62 indeed suppressed the proportions (Fig. 3i, j) and numbers (Table 1) of CD19^−^ B220^−^ CD138^+^ (from 8·31 to 3·69% live cells) and CD19^+^ B220^low/−^ CD138^+^ (from 1·37 to 0·72% live cells) plasma cells, which respectively are phenotypically similar to the long-lived plasma cell and short-lived plasma cell/plasmablast functional populations, reported previously.^16^–^18^ This presumably reflects reduced development and/or migration of such cells, as suggested by the significant increases in the levels of Fo1 (Fig. 2e) and CD19^−^ B220^−^ CD138^+^ plasma cells (numbers (× 10^6^) ± SEM: Naive, 0·75 ± 0·22; PBS, 1·28 ± 0·31; ES-62, 1·58 ± 0·26) found in the spleen, as ES-62 did not modulate the levels of early CD19^+^ B220^+^ CD138^+^ ‘pre-plasma cells’, which have been reported as being subject to a tolerance checkpoint that is defective in the autoimmune-prone MRL/Lpr mouse^19^ (results not shown).

**Table 1:** Exposure to ES-62 *in vivo* suppresses infiltration of the joints by B2 cells and plasma cells

Group	CD19^+^* *B220^low/*−*^* *CD138^+^		CD19^*−*^* *B220^*−*^* *CD138^+^		Fo1	
Exp	1	2	1	2	1	2
PBS	0·24	0·67	1·5	0·74	0·029	0·11
ES-62	0·035	0·32	0·18	0·36	0·009	0·033

Data (number of cells × 10^6^) are presented from two independent experiments where CD19^+^ B220^low/−^* *CD138^+^ and CD19^−^ B220^−^ CD138^+^ plasma cell and CD19^+^* *CD21^low^* *CD23^+^* *AA4·1^−^* *IgM^low^* *IgD^high^ Fol B-cell populations infiltrating the joints were analysed by flow cytometric analysis of joint cells harvested from six or seven mice from each group.

**Figure 4:**
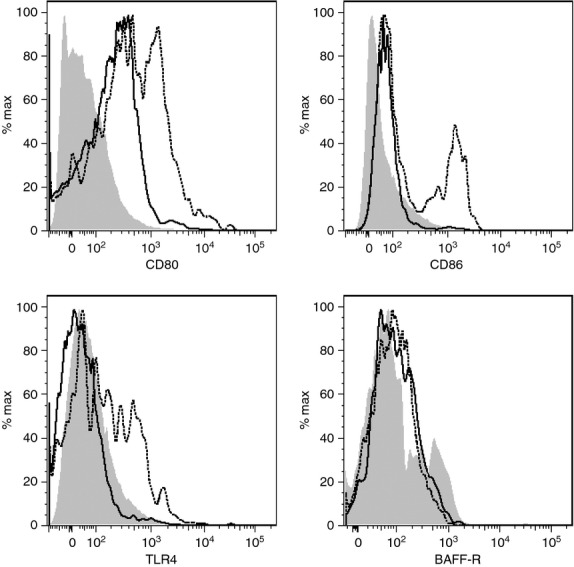
ES-62 modulates expression of Toll-like receptor 4 (TLR4) and co-stimulatory molecules on Follicular B cells. CD80, CD86, TLR4 and BAFF-R expression by CD19^+^* *CD23^high^ CD21^low^ follicular B cells in the joints of PBS and ES-62 treated mice are presented as expression levels relative to isotype control (grey area), for CIA mice treated with PBS (broken line) or ES-62 (black line). Cells from at least five mice/group were pooled.

In addition to investigation of modulation of the levels of B cells found in the joint, ES-62 was assessed for effects on the functional phenotype of such infiltrating cells. Hence, although ES-62 did not significantly modulate the expression of BAFF-R, CD80, CD86 or TLR4 (either in terms of percentage positive cells or levels of expression) on splenic CD19^+^ B cells, expression of CD80, TLR4 and, to a lesser extent, CD86, but not BAFF-R, by CD19^+^ B cells in the joint was down-regulated, mirroring a tendency towards a decrease in expression of both CD80 and TLR4 by CD19^+^ DLN cells (results not shown). Further analysis revealed that this reflected selective down-regulation of TLR4, CD80 and CD86 expression on CD19^+^* *CD23^high^ CD21^low^ Fo B cells but not CD19^+^* *CD23^−^* *CD21^high^ or CD19^+^* *CD23^high^* *CD21^high^ B cells (Fig. 4 and results not shown). Moreover, although B1, GC and plasma cells also expressed TLR4, such expression was not modulated by exposure to ES-62 (results not shown).

Collectively, therefore, these data suggest that ES-62 may act to prevent development and migration of pathogenic B cells to the site of inflammation, with the residual B cells that infiltrate the joint being rendered functionally hyporesponsive.

### ES-62 restores the levels of IL-10-producing B cells in the spleen of mice with CIA

**Figure 5:**
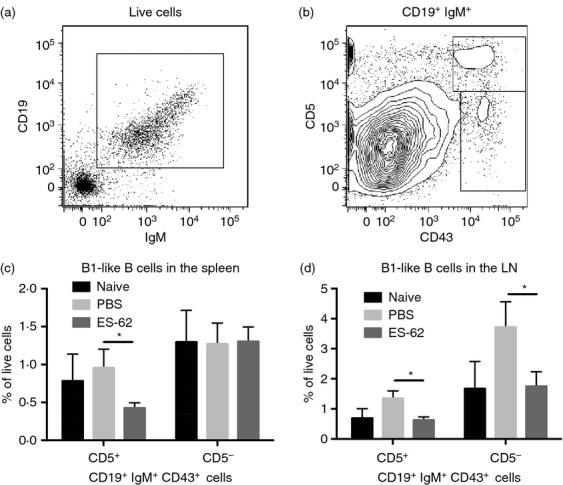
ES-62 modulates the levels of B1-like cells in the spleen and LNs of mice with collagen-induced arthritis (CIA). There is no unambiguous phenotype for B1 cells in the spleen but they have been described as CD19^high^ CD23^−^ CD43^+^ IgM^high^ IgD^low/−^ CD5^±^ cells where CD5^+^ B1a and CD5^−^ B1b comprise ˜2% and 1% of cells in the spleen, respectively.^24^ However, following gating on CD19^+^ IgM^+^ (a), analysis of CD43^+^ CD5^±^ cells has been widely used to describe B1 cells^25^,^26^ whereas CD19^+^ CD5^+^ gating has been used to describe B1a cells.^28^ Moreover, although CD43 can be up-regulated on B2 cells, this is usually expressed at a lower level than on B1 cells^27^ and we have therefore chosen to gate only on CD43^high^ cells (b) so as to exclude any potential CD43^+^ B2 cells. We have therefore phenotyped CD19^high^ CD43^+^ IgM^high^ B cells as CD5^+^ B1a-like cells and CD5^−^ B1b-like cells and the data show their relative proportions in the spleen (c; naive, *n* = 10; PBS, *n* = 13 and ES-62, *n* = 11) and draining lymph node (d; naive, *n* = 4; PBS, *n* = 8 and ES-62, *n* = 8) of the indicated groups of mice.

Interleukin-10-producing B cells that exhibit regulatory activity (Breg cells) have been reported to curb pathogenic Th1/Th17 responses in CIA and it has been proposed that these cells protect against disease by promoting the development of Tr1 cells.^6^ Although the ES-62-mediated suppression of B-cell development and migration (Figs 2 and 3) and consequent inhibition of generation of pathogenic IgG2a responses observed in CIA^11^ might reflect the ability of the parasitic worm product to induce hyporesponsiveness of B2 cells by uncoupling the B-cell receptor from extracellular signal regulated kinase/mitogen-activated protein kinase signalling, both *in vitro* and *in vivo*,^20^,^21^ it is also interesting to note that ES-62 has previously been shown to induce the production of IL-10 by peritoneal B1 B cells.^22^ As B1 cells are not thought to play a central role in systemic autoimmunity and autoantibody production in CIA,^23^ it is therefore possible that the reduced levels of CD19^+^ IgM^+^ CD43^high^ CD5^+^ and CD19^+^ IgM^+^ CD43^high^ CD5^−^ cells, which have been reported to be B1a and B1b cells, respectively,^24^–^28^ found in the spleens (Fig. 5a–c) and DLNs (Fig. 5d) of ES-62-treated mice with CIA could reflect their egress and migration to the joints to mediate IL-10-dependent anti-inflammatory effects. Perhaps consistent with this, although the levels of B cells infiltrating the joint overall were reduced following treatment with ES-62, the proportion of such B1-like cells was slightly increased (PBS: 1·42%, 1·3%; ES-62: 1·58%, 1·51%, where data are expressed as the proportion (%) of live cells, that are CD43^+^ CD19^+^ IgM^+^ B1-like cells, harvested from the joints of six or seven mice/group in two independent experiments).

**Figure 6:**
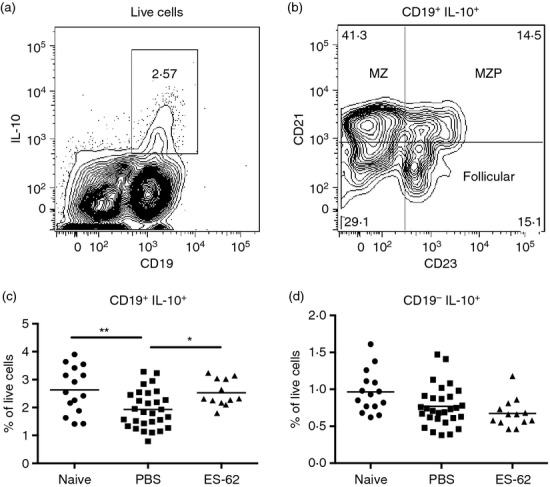
ES-62 induces interleukin-10 (IL-10)-producing B cells in the spleen of mice with collagen-induced arthritis (CIA). IL-10-producing CD19^+^ B cell (a,c) and IL-10-producing CD19^−^ non-B-cell subsets (a,d) in the spleen were analysed with the proportions of these cells, in spleens of individual naive mice and PBS- and ES-62-treated mice with CIA, shown respectively. Representative plots of the phenotypes of CD19^+^ IL-10^+^ B cells based on their expression of CD21 and CD23 (b) are shown for spleens of mice with CIA.

Analysis of IL-10-producing B cells (Fig. 6a,b) revealed that although induction of CIA resulted in a significant reduction in the levels of IL-10-producing B cells, relative to naive mice, this was not the case for those treated with ES-62 (Fig. 6c). Indeed, exposure to ES-62 led to a significant enhancement in the level of IL-10-producing B cells in mice with CIA, restoring them to levels comparable with those found in healthy naive mice [Fig. 6c; numbers (× 10^6^) ± SEM: Naive, 1·81 ± 0·22; PBS, 1·48 ± 0·12; ES-62, 1·99 ± 0·26]. CD19^+^ IL-10^+^ B cells in the spleens of naive and CIA mice reflected a mixed population comprising phenotypes consistent with marginal zone precursor, marginal zone, follicular B cells and CD19^+^ CD21^−^ CD23^−^ B cells (Fig. 6b): almost all of these cells expressed CD1d whereas some 20–30% of the follicular and CD19^+^ CD21^−^ CD23^−^ B cells expressed CD5 (data not shown), the latter a marker previously associated with certain IL-10-producing B cells.^29^,^30^ Rather surprisingly, the ability of ES-62 to return the levels of IL-10-producing B cells towards that existing in naive, non-arthritic mice, did not appear to involve a preferential modulation of any of these phenotypes, perhaps suggesting that it acts rather to regulate B-cell responses in a ‘homeostatic’ manner. Although the protective effects of IL-10-producing B cells in CIA have previously been reported to be associated with the induction of Tr1 cells,^6^ our analysis showed the IL-10 production by splenocytes to be predominantly B-cell-derived (Fig. 6a) and that the CD19^−^ IL-10^+^ population was not increased by treatment with ES-62 (Fig. 6d), suggesting that Tr1 cells were not being induced in this case. Likewise, and consistent with the previously reported lack of Breg-mediated induction of Treg cells in the CIA model,^6^ further investigation indicated that *in vivo* treatment with ES-62 did not result in enhanced levels of FoxP3-expressing CD4^+^ Treg cells in the DLN (results not shown), as shown following induction of Breg cells in AIA.^31^

## Discussion

The successful clinical implementation of B-cell depletion therapies in recent years has reignited interest in the pathogenic and protective roles of B cells in RA. In particular, much interest has focused on the ability of IL-10-producing Breg cells to prevent development of pathogenic Th1/Th17 responses and to induce Treg cells that result in the suppression of disease in the AIA (induced by methylated BSA) and CIA mouse models of RA.^6^,^8^,^31^ Interestingly, therefore it has recently emerged that one of the strategies exploited by parasitic helminths to dampen host immune responses and hence promote their survival is the induction of IL-10-producing Breg cells: moreover, the protection afforded against allergic inflammatory disease by such worms has also been associated, at least in part, with their induction of such Breg cells.^28^,^32^–^35^ Hence, our current findings that exposure to ES-62 results in elevated levels of IL-10-producing B cells may provide a rationale for our previous findings that the parasite product exerts its protective effects in CIA via suppression of Th1, Th17 and IL-17-producing *γδ* T-cell responses, as well as being consistent with our earlier observation that it promotes spontaneous IL-10 production by splenocytes from mice with CIA.^4^,^9^,^11^ Interestingly, although regulatory B cells have been proposed to mediate at least some of their protective effects in experimental arthritis via the generation of natural Treg cells (AIA) and/or induced Tr1 cells (AIA and CIA),^6^,^8^,^31^ we have found no evidence that ES-62 induces any IL-10-producing Treg cells in DBA/1 mice with CIA. Although perhaps surprising, this failure to induce Treg cells is supported by our preliminary data from the C57BL/6 model of chronic CIA,^36^ in which *in vivo* exposure to ES-62 does not increase the levels of either FoxP3^+^ or IL-10-producing CD4^+^ T cells in the DLN of such mice (results not shown) and is consistent with their lack of induction in our previous studies investigating ES-62-mediated hyporesponsiveness to the model antigen ovalbumin in both the DO.11.10 transfer model^37^ and the ovalbumin-induced airway inflammation model of asthma.^38^ Nevertheless, we cannot rule out the possibility that while ES-62 does not increase the levels of Treg cells in mice with CIA, it may act to reverse/overcome the impaired (cell contact-mediated) suppressive ability of Treg cells reported in RA.^39^

Perhaps also surprisingly, the restoration of IL-10-producing B cells resulting from exposure to ES-62 did not reflect induction of a particular phenotype of B cells associated with regulatory function: although modest, these statistically significant increases in IL-10-producing B cells are consistent with the numbers seen in other studies in the absence of enrichment by anti-CD40 or lipopolysaccharide/IL-21 stimulation^12^,^40^,^41^ and suggest that ES-62 may be acting in a homeostatic manner to reset the balance of effector and regulatory B cells back towards that observed in healthy DBA/1 mice. However, although the ES-62-mediated suppression of the level of GC B cells was associated with a reduction of follicular helper T cells, our preliminary data suggest that the residual follicular helper T cells produced slightly higher levels of the cytokine IL-21, which appears to be critical for the generation of functional Breg cells that combat autoimmunity.^41^ Hence, as we have not formally demonstrated the regulatory function of these IL-10-producing B cells, we cannot rule out the possibility at this stage that ES-62 is also reversing the defective regulatory function of one or more Breg phenotypes observed in CIA and also in RA patients.^42^,^43^ Nevertheless, we have shown previously that whereas ES-62 induces high levels of IgG1, but not IgG2a, antibodies in naive wild-type BALB/c mice, in IL-10^*−*/*−*^ mice the helminth product induces both IgG1 and IgG2a antibodies. This suggests that IL-10 plays an important role in the suppression of IgG2a antibodies directed against ES-62.^44^ Interestingly, therefore, we have shown that neither the ES-62-mediated suppression of DC-priming of OVA-specific Th1 responses^45^ nor the suppression of TLR-mediated IL-12 responses of macrophages exposed to ES-62 either *in vitro* or *in vivo*^46^ is due to autocrine production of IL-10 by APC. By contrast, although we have shown that exposure to ES-62 *in vivo* induces hyporesponsiveness of splenic B2 cells, peritoneal B cells from such mice produced enhanced spontaneous and B-cell receptor-stimulated IL-10 responses^21^,^22^ and although these peritoneal cells will predominantly comprise B1 cells, there is increasing evidence that they probably also include some B2 cells.^27^

Collectively, therefore, these data suggest that exposure to ES-62 leads to hyporesponsiveness of effector B2-cell responses and restoration of IL-10-producing B cells that in CIA, given the complex interplay between B-cell-derived IL-10 and pathogenic IL-17 responses in the regulation of inflammation and autoantibody responses,^6^,^12^,^31^,^41^,^47^–^49^ results in the reduction of plasma cells that may contribute to the suppression of pathogenic autoantibodies and inflammation associated with the protection against CIA. For example, the ability of B-cell-derived IL-10 to impact on antigen-presenting cells such as dendritic cells *in vivo* may contribute to the effects of ES-62 on the DC-dependent priming of Th1/Th17 and IL-17-producing *γδ* T cells.^4^,^38^,^45^ Alternatively, such IL-10 may suppress effector B-cell activation with consequent induction of T-cell hyporesponsiveness, as the reduction of GC B cells and follicular helper T cells observed in the spleens of ES-62-treated mice is reminiscent of that reported for mast cell-derived IL-10-mediated suppression of follicular helper T-cell function.^50^ This could be particularly important at the site of inflammation with the profound down-regulation of CD80 and CD86 expression on follicular B cells impacting on the functionality of ectopic GCs given that expression of CD80 by B cells has been shown to be important in the regulation of follicular helper T-cell development, and consequent GC B-cell survival and plasma cell production,^51^ the latter population being clearly reduced in the joints of ES-62-treated mice. Interestingly therefore, blocking of B7–CD28 interactions has been reported to be sufficient to prevent development of CIA.^52^ Finally, the strong down-regulation of TLR4 is similarly likely to suppress B-cell activation and plasma cell generation at this site,^53^ and in this way, disrupt the destructive chronic inflammation resulting from cells in the joint expressing up-regulated levels of TLRs, including TLR4^54^ and responding to damage-associated molecular pattern molecules, such as heat-shock protein 22 and tenascin-C^55^ found in the synovium of RA patients.

## Disclosures

The authors have no conflict of interest.

## Contributions

DTR, MAP, MAM and LA planned and performed experiments and WH and MMH planned and supervised the study. All authors contributed to the analysis of the data and preparation of the manuscript.

## Funding

This study was supported by research grants from the Wellcome Trust (WT086852) and ARUK (18413). DTR held a Wellcome Trust PhD studentship and MAM held an Oliver Bird/Nuffield Foundation PhD studentship.
